# Dr. Edmund Henry Kyle

**Published:** 1914-08

**Authors:** 


Dr. Edmund Henry Kyle, Pennsylvania, 1876, died at his home in Ithaca, July 9, aged 65.

